# Response of Key Metabolites during a UV-A Exposure Time-Series in the Cyanobacterium *Chlorogloeopsis fritschii* PCC 6912

**DOI:** 10.3390/microorganisms9050910

**Published:** 2021-04-24

**Authors:** Bethan Kultschar, Ed Dudley, Steve Wilson, Carole Anne Llewellyn

**Affiliations:** 1 Department of Biosciences, Swansea University, Singleton Park, Swansea SA2 8PP, UK; b.kultschar@outlook.com; 2 Swansea University Medical School, Swansea University, Singleton Park, Swansea SA2 8PP, UK; E.Dudley@Swansea.ac.uk; 3 Unilever R&D, Colworth Science Park, Sharnbrook, Bedfordshire MK44 1LQ, UK; Steve.Wilson@unilever.com

**Keywords:** Cyanobacteria, *C. fritschii*, GC-MS, metabolites, metabolomics, ultraviolet radiation, biotechnology

## Abstract

Ultraviolet A (UV-A) is the major component of UV radiation reaching the Earth’s surface, causing indirect damage to photosynthetic organisms via the production of reactive oxygen species (ROS). In comparison, UV-B causes both direct damage to biomolecules and indirect damage. UV-B is well studied in cyanobacterial research due to their long evolutionary history and adaptation to high levels of UV, with less work on the effects of UV-A. In this study, the response of key metabolites in *Chlorogloeopsis fritschii* (*C. fritschii*) during 48 h of photosynthetically active radiation (PAR, 15 µmol·m^−2^·s^−1^) supplemented with UV-A (11 µmol·m^−2^·s^−1^) was investigated using gas chromatography- mass spectrometry (GC-MS). Results showed an overall significant increase in metabolite levels up to 24 h of UV-A exposure. Compared with previously reported UV-B (PAR + UV-B) and PAR only results, UV-A showed more similarity compared to PAR only exposure as opposed to supplemented UV-B. The amino acids glutamate, phenylalanine and leucine showed differences in levels between UV (both supplemented UV-A and supplemented UV-B) and PAR only (non-supplemented PAR), hinting to their relevance in UV stress response. The fatty acids, palmitic and stearic acid, showed positive log2 fold-change (FC) in supplemented UV-A and PAR only experiments but negative log2 FC in UV-B, indicating the more harmful effect of UV-B on primary metabolism. Less research has been conducted on UV-A exposure and cyanobacteria, a potential environmental stimuli for the optimisation of metabolites for industrial biotechnology. This study will add to the literature and knowledge on UV-A stress response at the metabolite level in cyanobacteria, especially within the less well-known species *C. fritschii*.

## 1. Introduction

Cyanobacteria are known to survive in extreme environments and have adaptive mechanisms to cope with these conditions due to their evolution over 3.5 billion years [1]. During this time, they have been exposed to an array of abiotic factors such as an absence of the ozone layer in the Precambrian era, which led to the adaptation to high solar ultraviolet (UV) radiation [2]. Sunlight reaching the Earth’s surface (terrestrial and marine environments) consists of UV-A (315–400 nm), UV-B (280–315 nm), photosynthetically active radiation (PAR, 400–700 nm) and infrared radiation (IR, >700 nm). The majority of UV that reaches the Earth comprises of UV-A (approx. 95%) [3] with most solar UV-B absorbed by the ozone layer. 

UV-A differs from UV-B as it has a longer wavelength and is, therefore, lower in energy; unlike UV-B, it does not directly damage cells by reacting with biomolecules such as DNA and RNA, but causes damage indirectly via the production of reactive oxygen species (ROS) or energy transfer from UV-A-stimulated chromophores to target biomolecules such as DNA [4]. Cyanobacteria have many protective strategies to counteract the effects of UV radiation including avoidance, programmed cell death, DNA repair and production of the photoprotective compounds, mycosporine like-amino acids (MAAs) [4,5]. They also produce antioxidants which counteract the oxidative damage caused by ROS [6].

With the depletion of the ozone layer, much research has been conducted on the effect of elevated levels of UV-B on a variety of photosynthetic organisms [7,8,9]. Less research has been undertaken on UV-A as the intensity is not affected by ozone level changes [10].

There is much interest in the use of abiotic stressors such as UV-B in cyanobacterial research to induce protective mechanisms within cyanobacteria, to enhance the production of secondary metabolites which have potential for industrial purposes [11,12]. The majority of this research focuses on targeted metabolomic analysis of the photoprotective compounds MAAs investigated in; *C. fritschii* PCC 6912 [13,14], *Nostoc* sp. R76DM [15], *Leptolyngbya* sp. [16], *Anabaena* sp., *Nostoc commune* and *Scytonema* sp. [17] to name a few. Fewer studies have focused on untargeted metabolomic analyses for wider profiling of metabolites produced and affected by UV, but examples include metabolite level changes in *Nostoc flagelliforme* to different intensities of UV-B [18] and a proteomic and metabolomics investigation of *Nostoc punctiforme* ATCC 29133 during UV-A shock and short-term stress [19].

Metabolite profiling can be used to view the physiological changes at the metabolite level in response to changing environments, and is a useful tool in cyanobacterial research [20]. Furthermore, manipulating abiotic stress such as UV radiation can be used to enhance production of useful metabolites for uses in industrial biotechnology [11,12,21]. 

The main objective of this study was to analyze the changes in metabolite levels within the filamentous cyanobacterium *Chlorogloeopsis fritschii (C. fritschii*) PCC 6912, during supplemented UV-A (UV-A + PAR) exposure over a time-series of 48 h using gas-chromatography-mass spectrometry (GC-MS). Results were then compared to a previously reported study which used supplemented UV-B (UV-B + PAR) and PAR only exposure [14]. 

## 2. Materials and Methods

### 2.1. Organism and Growth Conditions

The cyanobacterium *C. fritschii* PCC 6912, (Pasteur Culture Collection) was maintained as previously described [14]. Briefly, cultures were maintained and grown in autoclaved deionised water supplemented with filtered BG-11 media (Sigma Aldrich) in a controlled temperature room at 27 ± 2 °C, under continuous PAR (Slyvania Gro-lux fluorescent tube) at 15 µmol·m^−2^·s^−1^ (measured using a PAR light sensor, Enviromonitors, UK). Stock cultures were grown in 300 mL BG-11 prior to experimental analysis under the same conditions with constant shaking at 80 rpm. 

### 2.2. Experimental Design

#### 2.2.1. Supplemented UV-A (UV-A + PAR) Exposure 

*C. fritschii* cultures (*n* = 3), pre-grown for 6 days, and transferred at an optical density at 750 nm (OD_750nm_) of approx. 0.16 into Quartz Erlenmeyer flasks (H.Baumbach & CO.LTD, Suffolk). UV-A was supplied by a Philips TL-D 18 W black light blue fluorescent tube (315–400 nm, centred at 360 nm) emitting 11 µmol·m^−2^·s^−1^ (measured using a UV light sensor, Enviromonitors, West Sussex, UK). A time-series experiment was carried out using the same conditions as above with constant shaking at 100 rpm for uniform UV-A exposure of cells. 

#### 2.2.2. Time-Series Sampling

UV-A-exposed cultures (*n* = 3) were harvested (40 mL) at 0 h (no UV-A), 2, 6, 12, 24 and 48 h by centrifugation (4400 rpm for 20 min) and the supernatant removed. The centrifuged pellets were immediately cryofrozen and stored at −80 °C until the end of the experiment. Pellets were then freeze-dried for 24 h (Scanvac, CoolSafe^TM^, LaboGene^TM^, Vassingerød, Denmark) and dry weight was measured. Dried pellets were stored at −20 °C for less than a month before analysis of intracellular metabolite levels (GC-MS) and carotenoid content (UV-visible spectroscopy). 

#### 2.2.3. GC–MS Analysis of Intracellular Metabolites

GC-MS analysis including sample preparation, derivatisation, data acquisition and processing, identification and statistical analysis was carried out as previously described [14]. Briefly, approx. 0.5–0.9 mg (UV-A exposed) dried biomass was extracted in methanol:chloroform:water (2:2:1) and sonicated (Fisher Scientific, FB50, 6 cycles of 20 s pulses at 40 Hz using an ice bath). 100 µL of each solvent layer was evaporated (Eppendorf concentrator 5301) and derivatised using methoxyamine hydrochloride (23 mg) in pyridine (1.5 mL) heated to 70 °C for 45 min followed by MSTFA + TMCS (Thermo Scientific^TM^, product no: TS-48915) for 90 min at 40 °C with tetracosane in hexane (2 mg/mL) as an internal standard. 

Derivatised samples were separated using an Agilent HP-5MS capillary column (30 m × 0.25 mm × 0.25 um) and data acquisition included a mass range of 50 to 650 *m*/*z*. Chromatograms were deconvoluted using Automated Mass Spectral Deconvolution and Identification System (AMDIS) followed by alignments using SpectConnect, http://spectconnect.mit.edu/ (accessed on 22 April 2021) [22,23], before identifying peaks using Golm metabolome database, http://gmd.mpimp-golm.mpg.de/ (accessed on 22 April 2021), and the National Institute of Standards and Technology 05 (NIST) library [24]. MetaboAnalyst, www.metaboanalyst.ca/ (accessed on 22 April 2021), was used for multivariate statistical analysis [25,26]. A two-sample *t*-test with equal variance was used for univariate statistical analysis, comparing no UV-A (0 h) with each time point (2, 6, 12, 24 and 48 h) of each detected peak in Excel 2010 (Microsoft, Redmond, WA, USA). Missing data points were assumed to be lower than the detection limit and were replaced with half of the minimum integrated signal for statistical analysis. Log2 fold-change (FC) was used to evaluate changes between 0 h and each time point (2, 6, 12, 24 and 48). 

Results were then compared to a previously reported study which used supplemented UV-B (UV-B + PAR) and PAR only exposure [14]. An analysis of variance (ANOVA) single factor was used to compare log2FC between treatments (UV-A + PAR vs. UV-B + PAR vs. PAR only) followed by Tukey’s honestly significant difference (HSD) test to determine the cause of significance.

#### 2.2.4. Carotenoid Analysis

Carotenoid analysis was carried out as previously described [14]. Briefly, approx. 0.5–0.9 mg of dried biomass was extracted in 100% HPLC grade methanol (1 mL) and absorbance spectra of supernatant was measured using a UV-visible spectrophotometer between (400–800 nm). Carotenoid concentration was calculated using the equations as described in [27,28].

## 3. Results

### 3.1. Intracellular Metabolite Levels during UV-A Exposure

Intracellular metabolite levels within *C. fritschii* were investigated during 48 h of UV-A exposure using GC-MS analysis. After data processing, the peaks were aligned in SpectConnect to yield a total of 210 detected peaks present throughout the 48 h time-series. A total of 113 of those showed statistical significance comparing between time points (one-way repeated ANOVA), with 16 remaining significant after Bonferroni correction. 

Of the 210 peaks detected, 175 were classified as ‘true-hits’, with 91 putatively identified (level 2; identified due to similarity to online spectra [29]) and 84 remaining unassigned (level 4; unknown [29]) using a match factor of ≥60% comparing to online spectra (Table S1). 

Comparing control (0 h) and each time point using a two-sample *t*-test, 24 h of UV-A had the highest statistical significance with 115 peaks (out of 175) and, of those, 84 showed a positive log2FC and 31 a negative log2FC (Table 1). Six hours of UV-A had the highest percentage (78%) of statistically significant peaks increasing in levels (81 with a positive log2FC). After 48 h of UV-A exposure a larger percentage (57%) of significant peaks showed a reduction in normalised abundance (34 with a negative log2FC). 

**Figure 1:**
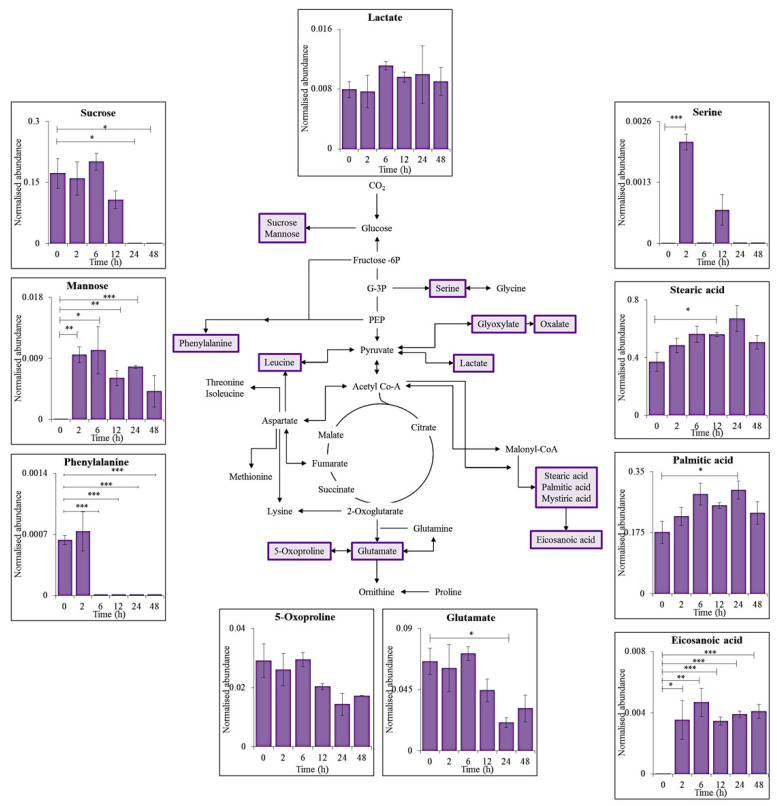
Schematic representation of a generalised reduced carbon metabolism in the cyanobacterium *C. fritschii.* Ten primary intracellular metabolites were identified using GC-MS and are highlighted in purple, with each graph presenting mean values of normalised abundance (normalised to internal standard and dry weight) ± standard error of each metabolite during supplemented UV-A exposure (UV-A + PAR). Statistical significance between control (0 h) and UV-A exposure (2, 6, 12, 24 and 48 h) was measured using a two-sample *t*-test with equal variance; * = 0.05 ≥ *p* > 0.01, ** = 0.01 ≥ *p* > 0.001 and *** = *p* ≤ 0.001.

Twenty-nine metabolites (10 shown for simplicity in Figure 1) were identified throughout the time-series known to have roles as primary metabolites within cyanobacterial metabolism including: glycolysis, the citric acid (TCA) cycle, amino acid, and fatty acid biosynthesis. 

The four amino acids, glutamate (Glu), leucine (Leu), serine (Ser) and phenylalanine (Phe) were identified with a match factor of ≥60%. A statistical decrease in Leu and Phe levels was seen after 6 h (*p* < 0.01 and *p* < 0.001, respectively) of supplemented UV-A exposure (UV-A + PAR), with Glu decreasing significantly after 24 h (*p* < 0.05). Ser levels significantly increased after 2 h of UV-A (*p* < 0.01). 

Four saturated fatty acids were also putatively identified, 3 of which showed significant increases after 2 h (eicosanoic acid, *p* < 0.05), 12 h (stearic acid, *p* < 0.05) and 24 h (palmitic acid, *p* < 0.05) of UV-A exposure, with mystiric acid levels showing steady levels with no significant changes (*p* > 0.05). 

The sugars mannose and sucrose were identified with significance during UV-A exposure, with sucrose levels decreasing significantly (0 vs. 24 h, *p* < 0.05) and mannose increasing significantly (0 vs. 2 h, *p* < 0.01) during the time-series. 

**Figure 2:**
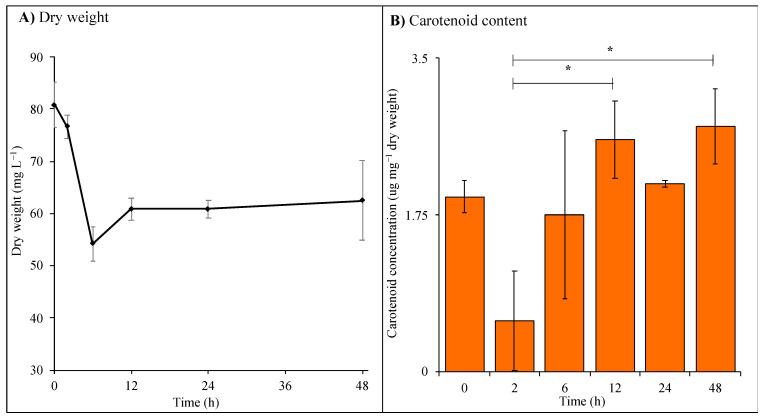
(**A**) Dry weight (mg·L^−1^) and (**B**) carotenoid content (µg·mg^−1^ dry weight) of *C. fritschii* cultures (*n* = 3) during supplemented UV-A exposure over 48 h. Statistical significance was calculated comparing control (0 h) and each time point (2, 6, 12, 24 and 48 h) using a two-sample *t*-test, * = 0.05 ≥ *p* > 0.01.

The dry weight measured decreased significantly to an average of 54.2 mg·L^−1^ after 6 h of supplemented UV-A exposure (*p* < 0.01, 0 vs. 6 h) with an increase up to an average of 62.5 mg·L^−1^ after 48 h with no significance measured (Figure 2A). Total carotenoid content (Figure 2B) decreased after 2 h of UV-A exposure followed by an increasing trend up to 48 h of exposure. 

### 3.2. Comparison of Supplemented UV-A, UV-B and PAR Only

**Figure 3:**
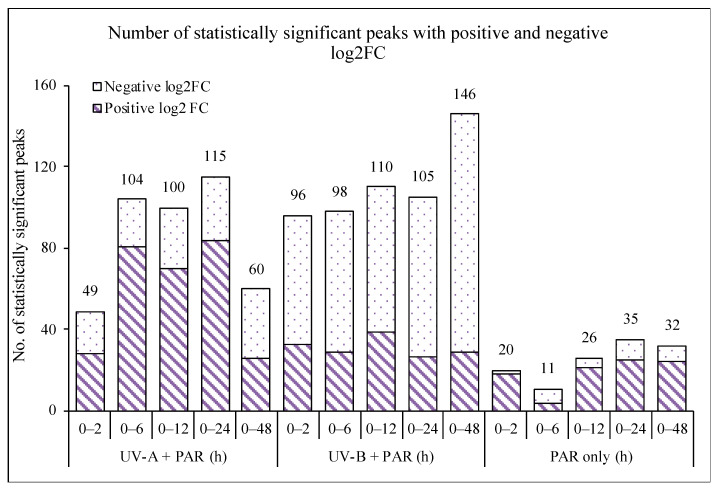
Total number of statistically significant peaks (*p* < 0.05) with positive and negative log2 Fold Change (FC) comparing control (0 h) to each time point 2, 6, 12, 24 and 48 h of UV exposed (UV-A + PAR or UV-B + PAR) and PAR only experiments. Results for UV-B +PAR from [14]. Significance was calculated using a two-sample *t*-test. Diagonal stripes represent positive log2FC and spots represent negative log2FC.

By comparing the UV-A results presented in this paper with a previously reported supplemented UV-B (UV-B + PAR) and PAR only experiment [14], differences were observed. PAR only had the lowest number of statistically significant peaks across the time-series (Figure 3) comparing 0 h to each time point but showed the highest percentage of positive log2FC (Table 2).

Supplemented UV-B showed an increasing number of significant peaks with increasing length of UV-B, with the majority of significant peaks decreasing in abundance (negative log2FC). Forty-eight (48) hours of UV-B showed the highest level of significance with 146 significant peaks; of those, 117 showed negative log2FC compared to the 63 decreasing in normalised abundance after 2 h of UV-B (Table 3). UV-A showed the majority of significant peaks increasing in metabolite levels (positive log2FC, Table 1).

**Figure 4:**
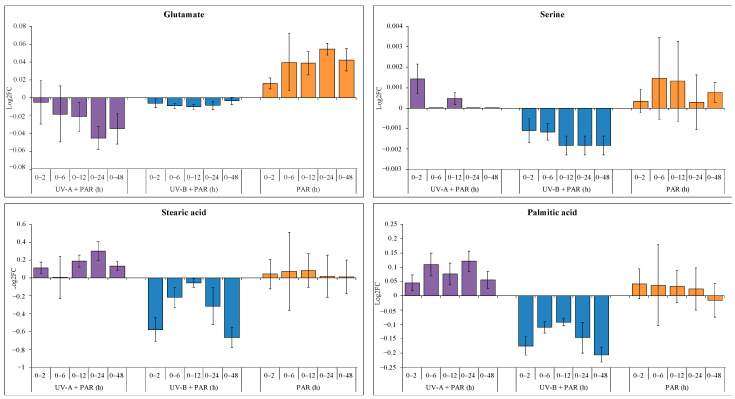
Log2FC of commonly identified metabolites during supplemented UV-A (purple), supplemented UV-B (blue) and PAR only (orange) exposure, comparing 0 h and each time point (2, 6, 12, 24 and 48 h). A positive log2FC indicates an increase in metabolite levels, and a negative log2FC shows a decrease in metabolite levels.

Eight (8) common metabolites were identified between supplemented UV-A, supplemented UV-B and PAR only exposure. These included Glu, Ser, palmitic acid, and stearic acid. Next, using a one-way ANOVA-single factor, comparisons of log2FC of the common metabolites (control (0 h) vs. each time point) between the three treatments were investigated (Figure 4, Table S2). 

Glu showed significance between the three treatments when comparing log2FC at 0 vs. 12 h (F (2, 6) = 6.77, *p* < 0.05), 0 vs. 24 h (F (2, 6) = 32.70, *p* < 0.001) and 0 vs. 48 h (F (2, 6) = 9.60, *p* < 0.05) with the post hoc Tukey’s HSD test showing significance between supplemented UV-A and PAR only at 0 vs. 12 h (*p* = 0.031), 0 vs. 24 h (*p* = 0.001) and 0 vs. 48 h (*p* = 0.011), as well as supplemented UV-B and PAR only at 0 vs.24 h (*p* = 0.005). No significance was observed between UV-A and UV-B. Ser showed significance comparing log2FC of 0 vs. 48 h (F (2, 6) = 11.86, *p* < 0.01). This significance was assessed using a Tukey’s HSD test and significance was found when comparing treatments to UV-B (UV-A vs. UV-B, *p* = 0.035; PAR vs. UV-B, *p* = 0.008), with no significance comparing supplemented UV-A and PAR.

Palmitic acid showed significance between the three treatments comparing 0 vs. 2 h (F (2, 6) = 10.59, *p* < 0.05), 0 vs. 24 h (F (2, 6) = 5.79, *p* < 0.05) and 0 vs. 48 h (F (2, 6) = 10.89, *p* < 0.05). The Tukey’s HSD test results showed no significance between log2FC of UV-A and PAR only exposure experiments, but significance was observed between UV-A and UV-B (0 vs. 2 h, *p* = 0.016; 0 vs. 24 h, *p* = 0.035; 0 vs. 48 h, *p* = 0.0096) as well as UV-B and PAR (0 vs. 2 h, *p =* 0.017; 0 vs. 48 h, *p* = 0.038). Stearic acid shows significance at 0 vs. 2 h (F (2, 6) = 9.71, *p* < 0.05) and 0 vs. 48 h (F (2, 6) = 10.22, *p* < 0.05). The Tukey’s HSD test revealed significance due to UV-B (UV-A vs. UV-B [0 vs. 2 h, *p* = 0.02; 0 vs. 48 h, *p* = 0.012] and PAR only vs. UV-B [0 vs. 2 h, *p* = 0.021; 0 vs. 48 h, *p* = 0.037]).

**Figure 5:**
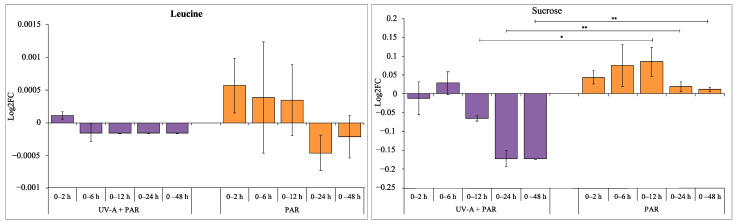
Log2FC of commonly identified metabolites during supplemented UV-A (purple) and PAR only (orange) exposure, comparing 0 h and each time point (2, 6, 12, 24 and 48 h). A positive log2FC indicates an increase in metabolite levels, and a negative log2FC shows a decrease in metabolite levels. Statistical significance between log2FC values was calculated using a two-sample *t*-test, * = 0.05 ≥ *p* > 0.01, ** = 0.01 ≥ *p* > 0.001.

An additional two metabolites were identified in both supplemented UV-A and PAR only exposure: Leu and sucrose (Figure 5). Leu showed opposite log2FC patterns with supplemented UV-A, decreasing in normalised abundance compared to 0 h, with a general increasing trend of the normalised mean abundance during the PAR only time-series with no significance observed (*p* > 0.05) due to the high error of the PAR only data. Sucrose also showed a similar opposite pattern, with decreasing abundance and with increasing length of UV-A and increasing during PAR only exposure (0 vs. 12 h, *p* < 0.05; 0 vs. 24 h, *p* < 0.01 and 0 vs. 48 h, *p* < 0.01).

**Figure 6:**
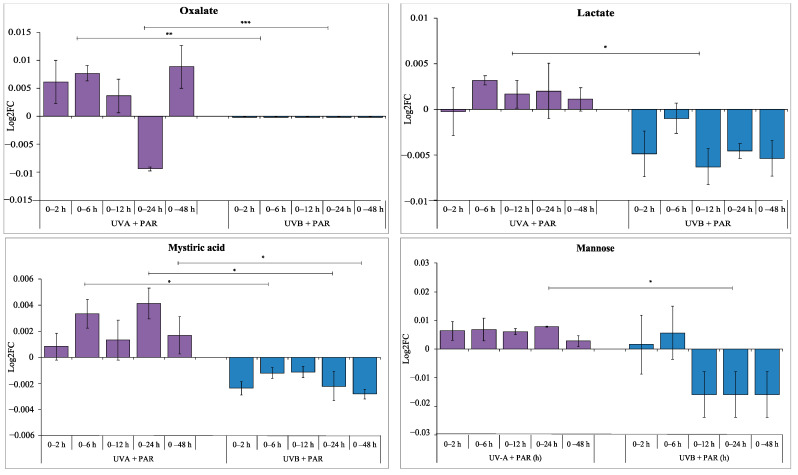
Log2FC of commonly identified metabolites during supplemented UV-A (purple) and UV-B (blue) exposure comparing no UV (0 h) and each time point (2, 6, 12, 24 and 48 h). A positive log2FC indicates an increase in metabolite levels, and a negative log2FC shows a decrease in metabolite levels. Statistical significance between log2FC values was calculated using a two-sample *t*-test, * = 0.05 ≥ *p* > 0.01, ** = 0.01 ≥ *p* > 0.001 and *** = *p* ≤ 0.001.

These opposite log2FC patterns were also observed between supplemented UV-A and supplemented UV-B time-series data (Figure 6) including: oxalate (0 vs. 6 h, *p* < 0.01; 0 vs. 24 h, *p* < 0.01*)*, lactate (0 vs. 12 h, *p* < 0.05), mystiric acid (0 vs. 6 h, *p* < 0.05; 0 vs. 24 h, *p* < 0.05 and 0 vs. 48 h, *p* < 0.05) and mannose (0 vs. 24 h, *p* < 0.05). 

## 4. Discussion

### Effects of UV Exposure on C. fritschii

The effect of UV-B on cyanobacteria is widely researched compared to the effect of UV-A. UV-A and UV-B have different effects on photosynthetic organisms and mechanisms of protection vary [4,10]. An increase in carotenoid levels (Figure 2B) was observed with increasing length of UV-A; this is consistent with previous experiments during UV-B exposure [14]. This accumulation could be due to the increased ROS production caused by increasing length of UV where carotenoids act as antioxidants. An initial decrease in dry weight (Figure 2A) indicates the shock response of *C. fritschii* to initial UV-A exposure. An increase in biomass was then observed between 6 h and 48 h showing the acclimation of cells to UV-A, as supported by the GC-MS data, where less significance is seen between 0 h and 48 h of supplemented UV-A (Table 1).

The metabolite Glu showed negative log2FC in both supplemented UV-A and UV-B compared to the positive log2FC trend during PAR only exposure (Figure 4), thus suggesting the role of this amino acid during UV stress. A decrease in Phe levels during UV-A exposure (Figure 1) is consistent with the need for aromatics such as phenylpropanoids which can absorb harmful UV due to their unsaturated nature, and for protein synthesis [30]. Glu is involved in glutathione synthesis, which has a role as an antioxidant and protects cells from ROS, an example of which includes the conversion of H_2_O_2_ into H_2_O to prevent the formation of •OH [31]. 

The soluble sugar sucrose, a disaccharide consisting of glucose and fructose, has been seen to have a role in stress tolerance in both plants and cyanobacteria [18,32] which is consistent with the opposite effect between UV-A exposure and PAR only exposure (Figure 5). 

UV-A and PAR only exposure have opposite effects on the saturated fatty acids stearic acid and palmitic acid compared to UV-B, with the accumulation during UV-A and PAR only and a decrease during UV-B exposure. This trend is also seen with mystiric acid (Figure 6, comparing UV-A and UV-B). Saturated fatty acids provide energy for the rebuilding of photosynthetic apparatus which could be damaged directly by UV-B but to a lesser extent during UV-A irradiation [18]. 

During this study we have been able to investigate the changes during supplemented UV-A exposure on *C. fritschii* cultures at the metabolite level. Overall, these results show that supplemented UV-A is less detrimental to *C. fritschii* cells compared to UV-B; significantly a higher proportion of metabolites increased in levels during UV-A exposure compared to a previously reported UV-B study. We also found that the metabolites Phe, Glu and sucrose are affected by both UV-A and UV-B associating them with UV stress response. Reductions in fatty acids, oxalate, mannose and lactate levels could be associated with UV-B stress response due to the direct and indirect damaging effects. 

This study can be used to build on the knowledge of the effect of UV on metabolites produced by C*. fritschii* and other cyanobacteria. To fully understand these mechanisms of stress response, more work needs to be carried out on a diversity of cyanobacterial species. By investigating a variety of different UV conditions and integrating multiple–omics techniques, the optimisation of useful metabolite production for industrial biotechnology can be achieved. 

## Figures and Tables

**Table 1:** Comparison of statistically significant (*p* < 0.05) peaks (out of 175) and number of those significant peaks with a positive or negative log2 fold change (FC). A positive log2FC indicates an increase in metabolite levels and a negative log2FC value indicates a decrease in metabolite levels. Both significance and log2FC were calculated comparing control (0 h) and 2, 6, 12, 24 and 48 h of UV-A exposure.

UV-A Exposure (h)	No. of Peaks, 12*p <* 0.05 ^a^	No. of Peaks, Positive log2FC	No. of Peaks, Negative log2FC
**0 vs. 2**	49	28	21
**0 vs. 6**	104	81	23
**0 vs. 12**	100	70	30
**0 vs. 24**	115	84	31
**0 vs. 48**	60	26	34

^a^ = Significance calculated using a two-sample *t*-test with equal variance comparing control (0 h) to each UV-A exposed time point.

**Table 2:** Comparison of statistically significant (*p* < 0.05) peaks (out of 137) and number of those significant peaks with a positive or negative log2FC during PAR only exposure. A positive log2FC indicates an increase in metabolite level and a negative log2FC value indicates a decrease in metabolite level. Both significance and log2FC was calculated comparing control (0 h) and 2, 6, 12, 24 and 48 h of PAR only exposure. PAR data are sourced from previous work [14].

PAR Only (h)	No. of Peaks, *p <* 0.05 ^a^	No. of Peaks, Positive log2FC	No. of Peaks, Negative log2FC
**0 vs. 2**	20	18	2
**0 vs. 6**	11	4	7
**0 vs. 12**	26	21	5
**0 vs. 24**	35	25	10
**0 vs. 48**	32	24	8

^a^ = Significance calculated using a two-sample *t*-test with equal variance.

**Table 3:** Comparison of statistically significant (*p* < 0.05) peaks and number of those significant peaks with a positive or negative log2FC during supplemented UV-B exposure. A positive log2FC indicates an increase in metabolite level and a negative log2FC value indicates a decrease in metabolite level. Both significance and log2FC was calculated comparing control (0 h) and 2, 6, 12, 24 and 48 h of UV-B exposure. UV-B data are sourced from previous work [14].

UV-B Exposure (h)	No. of Peaks, *p <* 0.05 ^a^	No. of Peaks, Positive log2FC	No. of Peaks, Negative log2FC
**0 vs. 2**	96	33	63
**0 vs. 6**	98	29	69
**0 vs. 12**	110	39	71
**0 vs. 24**	105	27	78
**0 vs. 48**	146	29	117

^a^ = Significance calculated using a two-sample *t*-test with equal variance.

## Data Availability

Data available by contacting the corresponding author.

## Supplementary Materials

The following are available online at https://www.mdpi.com/article/10.3390/microorganisms9050910/s1, Table S1: Intracellular peaks detected during UV-A exposure using GC-MS including; Retention time (RT), name, class, level, model (m/z), normalised mean abundance (*n* = 3 or 2) ± standard error (SE). *t*-test and ANOVA results showing statistical significant compounds, * = 0.05 ≥ p > 0.01, ** = 0.01 ≥ p > 0.001, *** = p ≤ 0.001, BF =significant after Bonferroni correction. Table S2: Primary intracellular metabolites detected during UV-A + PAR, UV-B + PAR and PAR only including possible biosynthetic pathways.
